#  Association of Medicare Advantage Star Ratings With Racial, Ethnic, and Socioeconomic Disparities in Quality of Care

**DOI:** 10.1001/jamahealthforum.2021.0793

**Published:** 2021-06-11

**Authors:** David J. Meyers, Momotazur Rahman, Vincent Mor, Ira B. Wilson, Amal N. Trivedi

**Affiliations:** 1 Department of Health Services, Policy, and Practice, Brown University School of Public Health, Providence, Rhode Island; 2 Providence VA Medical Center, Providence, Rhode Island

## Abstract

**Importance:**

Medicare Advantage (MA) plans, which disproportionately enroll racial/ethnic minorities and persons with socioeconomic disadvantage, receive bonus payments on the basis of overall performance on a 5-star rating scale. The association between plans’ overall quality and disparities in quality is not well understood.

**Objective:**

To examine the association between MA star ratings and disparities in care for racial/ethnic minorities and enrollees with lower income and educational attainment.

**Design, Setting, and Participants:**

This cross-sectional study included 1 578 564 MA enrollees from 454 contracts across the 2015 and 2016 calendar years. Data analyses were conducted between June 2019 and June 2020.

**Exposures:**

Self-reported race and ethnicity and low socioeconomic status (SES) (defined by low income or less than a high school education) vs high SES (neither low income nor low educational attainment).

**Main Outcomes and Measures:**

Performance on 22 measures of quality and satisfaction determined at the individual enrollee level, aggregated into simulated star ratings (scale, 2-5) stratified by SES and race/ethnicity.

**Results:**

A total of 1 578 564 enrollees were included in this analysis (55.8% female; mean [SD] age, 71.4 [11.3] years; 65.8% White; 12.3% Black; 14.6% Hispanic). Enrollees with low SES had simulated stratified star ratings 0.5 stars lower (95% CI, 0.4-0.6 stars) than individuals with high SES in the same contract. Black and Hispanic enrollees had simulated star ratings that were 0.3 stars (95% CI, 0.2-0.4 stars) and 0.1 stars (95% CI, −0.04 to 0.2 stars) lower than White enrollees within the same contracts. Black enrollees had a 0.4-star lower rating (95% CI, 0.1-0.7 stars) in 4.5- to 5-star contracts and a no statistical difference in 2.0- to 2.5-star–rated contracts (difference, 0.3 stars; 95% CI, −0.02 to 0.7 stars). Hispanic enrollees had a 0.6-star lower simulated rating (95% CI, 0.2-1.0 stars) in 4.5- to 5-star contracts and no statistical difference in 2- to 2.5-star contracts (difference, −0.01 stars; 95% CI, −0.5 to 0.4 stars). There was low correlation between simulated ratings for enrollees of low SES and high SES (difference, 0.2 stars; 95% CI, 0.03-0.4 stars) and between simulated ratings for White and Black enrollees (difference, 0.4 stars; 95% CI, 0.3-0.5 stars) and White and Hispanic enrollees (difference, 0.3 stars; 95% CI, 0.2-0.4 stars). As the proportion of Black and Hispanic enrollees increased in a contract, racial/ethnic disparities in ratings decreased.

**Conclusions and Relevance:**

In this cross-sectional study, simulated MA star ratings were only weakly correlated with those for enrollees of low SES in the same contract, and contracts with higher star ratings had larger disparities in quality. Measures of equity in MA plans’ quality of care may be needed.

## Key Points

**Question:**

Do Medicare Advantage star ratings, which are generated using data from all enrollees in a plan, reflect the quality experience of racial/ethnic minorities and enrollees with low socioeconomic status (SES)?

**Findings:**

This cross-sectional study of 1 578 564 Medicare Advantage enrollees found that simulated star ratings for persons with lower SES and Black and Hispanic enrollees were substantially lower than ratings for those with higher SES and White enrollees in the same contract. There was little correlation between ratings for each group, and higher-performing plans, as measured by the star rating, had larger racial/ethnic and SES disparities in care.

**Meaning:**

Medicare Advantage star ratings, which are designed to reflect overall performance in a plan, are only modestly associated with quality for racial/ethnic minorities and enrollees of low SES in the same plan.

## Teaser

This cross-sectional study examines the associations between Medicare Advantage star ratings and disparities in care for racial/ethnic minorities and enrollees with lower income and educational attainment.

## Introduction

More than one-third of Medicare beneficiaries were enrolled in Medicare Advantage (MA) in 2019.^1,2^ In MA, private plans receive capitated payments to cover their enrollees’ health care needs. Medicare Advantage plans enroll higher proportions of racial/ethnic minorities and enrollees with lower income and education than the traditional Medicare program, and prior work has found substantial disparities in the quality of care in the MA program.^3,4,5,6,7,8,9,10,11,12,13,14^ These disparities in care have been found within plans (disparities in quality of care for enrollees in the same plans), and between plans (disparities driven by disproportionate enrollment of minorities in plans with worse quality).^3,13,14^

Since 2008, the US Centers for Medicare & Medicaid Services (CMS) has used a 5-star rating system to measure the performance of MA contracts and allocates $6 billion in annual bonus payments on the basis of these star ratings. Then CMS calculates these ratings and assigns bonus payments for all enrollees in a contract without stratifying results by race, ethnicity, or socioeconomic status (SES). While the CMS Office of Minority Health reports some individual measures by race/ethnicity dual status, as well as by disability, this stratification does not contribute to plan payment decisions. If aggregate contract star ratings hide clinically important differences in quality between advantaged and disadvantaged plan members, then quality measures that directly assess equity may be needed.^15,16^ This study addressed 2 main points: first, if there is an association between an MA contract’s overall star rating and what the star rating would have been if calculated for that contract’s enrollees who are racial/ethnic minorities or who have lower SES; second, if contracts with higher star ratings have lower disparities in care.

## Methods

### Data Sources

We compiled individual-level data on 22 measures included in CMS’s current star-rating calculation using 4 primary sources from the 2015 and 2016 calendar years. First, we used the Medicare Health Outcomes Survey (HOS), which randomly samples 1200 enrollees from each MA contract.^17^ Five variables from HOS are used in the star rating (eTable 1 in the Supplement). We focused on measures included in MA star ratings and did not include measures that evaluate Part D benefits, as not all plans include Part D benefits. Second, we used the MA Consumer Assessment of Healthcare Providers and Systems (CAHPS), an annual cross-sectional survey of 600 enrollees from each MA contract.^18^ Third, we used 10 person-level measures from the MA Healthcare Effectiveness Data and Information Set files.^19^ We linked beneficiaries to the Master Beneficiary Summary File (MBSF) to identify their MA contract and to calculate our measure of disenrollment. We considered an enrollee as disenrolled from the plan if in the following year they were enrolled in a different MA plan or in traditional Medicare, excluding those who moved counties between years and those in plans that exited the MA market.

This study was approved by the institutional review board at Brown University and received a waiver of informed consent owing to use of deidentified data. Analyses took place between June 2019 and June 2020, and Strengthening the Reporting of Observational Studies in Epidemiology (STROBE) reporting guidelines were followed.

### Classifying SES and Race/Ethnicity

Both the CAHPS and HOS include questions on SES and self-reported race/ethnicity. We selected low income and less than high school education as measures of low SES; both have been identified by the National Academy of Medicine as key social determinants of health.^20^ We classified an individual as having low income if they were dually eligible (including both full and partial) with Medicaid or were eligible for the Part D low-income subsidy as specified in either the HOS, CAHPS, or MBSF.^21^ We identified individuals with less than a high school education based on self-reported questions in both surveys. We stratified the results by enrollees who had both low income and low education (both low SES) compared with those who had either low income or low education (either low SES) and those who had neither low income nor low education (high SES).

Both the CAHPS and HOS include self-reported race/ethnicity data. To align with CMS’s methodologies, we classified enrollees who self-reported their ethnicity as Hispanic as Hispanic, regardless of what race they selected. We classified an enrollee as non-Hispanic Black (hereafter Black) if they reported their race as Black in either the HOS or CAHPS and did not report Hispanic ethnicity. For the race/ethnicity comparisons, the comparison group comprised those who self-identified as non-Hispanic White. We report the agreement between the self-reported and MBSF designations in eTable 3 in the Supplement.

### Study Population

We included all beneficiaries who responded to the HOS and CAHPS surveys in 2015 and 2016 in our analysis and combined the years to provide a larger sample. We excluded enrollees who were enrolled in employee-sponsored MA contracts and in Medicare-Medicaid fully integrated plans. To ensure the stability of our analysis, we required that every contract have at least 50 sampled enrollees from each SES and race/ethnicity group. In sensitivity analyses, we also tested requiring at least 100 enrollees from each group to test the stability of our estimates. Response rates did not vary substantially by survey or study population (eTable 5 in the Supplement).

### Calculation of Simulated Star Ratings

To determine a star rating, CMS calculates each contract on the basis of 30 to 35 measures of satisfaction and quality outcomes.^22^ Then CMS assigns ratings at the contract level. Each contract may contain with it any number of plans that may vary in their benefits and cost sharing. We performed our analysis at the contract level, consistent with CMS’s rating methods (eMethods in the Supplement). We replicated CMS’s approach with 22, or 70%, of all the measures included in star-rating calculation based on 2016 cut points. When CMS calculates star ratings, the survey data and the cut points to assign different measures vary from year to year. In this study, we use 2016 cut points to estimate 2016 ratings; however, because these survey data are from across several years, our simulated ratings are not identical to those of CMS. We tested alterative methods for calculating the ratings using 2015 and 2017 cut points, and by averaging 2015, 2016, and 2017 cut points, but the results did not vary substantially. Our simulated overall star rating was highly correlated with the official 2015 and 2016 star ratings (both correlations = 0.9). After calculating a simulated overall star rating, we calculated stratified star ratings using only enrollees in each of the 5 stratified groups (high SES, low SES, White, Black, and Hispanic).

### Statistical Analysis

We calculated the difference between each contract’s simulated star rating for high-SES and low-SES groups, and between each contract’s White and Black or Hispanic enrollees. We compared differences in contract-level ratings for each of these groups using analysis of variance, χ^2^ statistical tests, and 95% CIs. Given the complexity of the different data sources that are included in this analysis, we calculated all SEs from bootstrapping with 10 000 replications. We calculated the sample correlation between measures using Pearson correlation coefficients.

We also decomposed any disparity in ratings into within-contract and between-contract differences following methods previously described in the literature.^3,23^ The within-contract difference was calculated as the contract-level difference in the rating of each group, averaged across all contracts. We calculated the between-contract difference by taking the mean of the contract-level differences weighted by the number of Black or Hispanic enrollees and enrollees of low SES in each contract. We then subtracted this weighted difference from the total difference to calculate the between disparity.^23^ The within-contract disparity can be interpreted as the disparity in outcomes attributable to differences in outcomes among persons enrolled within the same contract, while the between-contract disparity can be interpreted as the disparity in outcomes attributable to persons with low SES or racial/ethnic minorities being disproportionately enrolled in contracts with lower star ratings.

We intentionally did not include any adjustments from multivariable regression analysis in this study for 2 reasons. First, most measures included in CMS star-rating calculation do not use adjustment, and we wanted to simulate CMS’s methods. Second, while adjustment can help compare performance on a more even playing field, it may also obscure absolute differences in performance between groups. We elect to stratify the simulated results to better highlight where differences exist.

In sensitivity analyses, we compared the sample sizes included in each measure; compared results when excluding Puerto Rico, because the MA market and disparities within plans may function differently outside the continental US^9^; and compared absolute differences between simulated ratings and individual measures within contract. We also calculated the interunit reliabilities for each measure across contracts and strata to assess to what extent disparities between contracts may be related to chance (eTable 8 in the Supplement).^24,25,26^ All analyses were conducted in Stata, version 16 (StataCorp), and a 2-sided α = .05 was considered statistically significant.

## Results

Table 1 summarizes the demographic and contract characteristics of the 1 578 564 MA enrollees (55.8% female; mean [SD] age, 71.4 [11.3] years) included in the analysis, stratified by SES and race/ethnicity. Of the enrollees in the sample, 7.0% were of low SES, and 12.3% and 14.6% of the sample were Black and Hispanic, respectively.

**Table 1.** Demographic and Contract Characteristics of Study Population by Socioeconomic Status (SES) and Race/Ethnicity^a^

Characteristic	No. (%)					
	High SES	Low SES (low income or low education)	Low SES (low income and low education)	Race/ethnicity		
				White	Black	Hispanic
Total	985 204	523 913	69 447	1 012 303	200 057	217 917
Year of rating						
2015	530 095 (53.8)	292 700 (55.9)	39 168 (56.4)	553 176 (54.6)	108 501 (54.2)	119 199 (54.7)
2016	455 109 (46.2)	231 213 (44.1)	30 279 (43.6)	459 127 (45.4)	91 556 (45.8)	98 718 (45.3)
Age, mean (SD), y	73.0 (9.0)	68.6 (14.2)	71.1 (11.9)	72.3 (10.8)	68.0 (12.6)	70.2 (11.7)
Sex						
Female	525 411 (53.3)	314 580 (60.0)	42 104 (60.6)	558 864 (55.2)	120 419 (60.2)	119 453 (54.8)
Male	460 351 (46.7)	209 720 (40.0)	27 375 (39.4)	453 571 (44.8)	79 613 (39.8)	98 527 (45.2)
Race/ethnicity						
White	764 435 (77.6)	249 351 (47.6)	24 187 (34.8)	NA	NA	NA
Black	84 305 (8.6)	108 916 (20.8)	15 992 (23.0)	NA	NA	NA
Other^b^	19 479 (2.0)	7583 (1.4)	924 (1.3)	NA	NA	NA
Asian	28 445 (2.9)	35 424 (6.8)	4075 (5.9)	NA	NA	NA
Hispanic	86 378 (8.8)	120 053 (22.9)	23 980 (34.5)	NA	NA	NA
Native American/American Indian	2162 (0.2)	2586 (0.5)	289 (0.4)	NA	NA	NA
Dual eligibility with Medicaid	NA	420 771 (80.3)	59 372 (85.5)	188 294 (18.6)	94 805 (47.4)	101 357 (46.5)
<High school	NA	127 329 (24.3)	69 447 (100)	57 646 (5.7)	25 791 (12.9)	40 337 (18.5)
Low income	NA	466 031 (89.0)	69 447 (100)	211 854 (20.9)	105 005 (52.5)	111 713 (51.3)
**Plan characteristics**						
Type of plan						
HMO	641 832 (68.0)	423 658 (85.2)	58 298 (87.4)	657 507 (68.2)	161 806 (84.7)	184 172 (88.0)
PPO	283 759 (30.1)	67 728 (13.6)	7692 (11.5)	285 675 (29.6)	27 281 (14.3)	24 478 (11.7)
Other	17 808 (1.9)	5613 (1.1)	727 (1.1)	20 568 (2.1)	1897 (1.0)	643 (0.3)
Premium tertile						
1	151 108 (30.1)	77607 (28.2)	11 325 (30.3)	123 344 (23.8)	37 832 (36.9)	56 653 (49.8)
2	125 680 (25.1)	15 0751 (54.8)	21 234 (56.8)	169 148 (32.6)	46 150 (45.0)	41 647 (36.6)
3	224 537 (44.8)	46 502 (16.9)	4836 (12.9)	226 833 (43.7)	18 658 (18.2)	15 401 (13.5)
Star rating						
2.5	16 500 (2.6)	19 938 (5.5)	3496 (7.2)	18 281 (2.8)	8599 (6.9)	7721 (5.0)
3	78 910 (12.6)	98 900 (27.5)	13 887 (28.8)	84 723 (12.9)	40 519 (32.7)	41 028 (26.7)
3.5	170 371 (27.1)	111 035 (30.8)	14 435 (29.9)	178 614 (27.2)	31 950 (25.8)	50 685 (33.0)
4	189 617 (30.2)	81 898 (22.8)	10 742 (22.3)	194 168 (29.5)	27 385 (22.1)	36 343 (23.7)
4.5	148 233 (23.6)	42 692 (11.9)	5151 (10.7)	157 049 (23.9)	13 955 (11.3)	15 071 (9.8)
5	25 011 (4.0)	5523 (1.5)	526 (1.1)	24 812 (3.8)	1405 (1.1)	2650 (1.7)

Abbreviations: HMO, health maintenance organization; PPO, preferred provider organization; NA, not applicable.

^a^ Based on individual-level data from the Master Beneficiary Summary File, Consumer Assessment of Healthcare Providers and Systems, Medicare Health Outcomes Survey, and Healthcare Effectiveness Data and Information Set in 2015 and 2016. Individuals may be included in multiple low SES and race/ethnicity categories. Low SES refers to enrollees with low income and/or low education; low income is defined as being dually eligible for Medicaid or receiving the low-income subsidy, and low education is defined as having less than a high school education. The high SES category is defined as individuals who had neither low income nor low education. Data were only included for individuals who were sampled by at least the Consumer Assessment of Healthcare Providers and Systems or the Medicare Health Outcomes Survey.

^b^ Other denotes those whom the US Centers for Medicare & Medicaid Services classifies as Other Race/Ethnicity or Unknown Race/Ethnicity. No further detail is available in the Master Beneficiary Summary File.

Table 2 summarizes the mean simulated stratified star ratings at the contract level. Enrollees of low SES had a 0.5-star lower simulated rating than enrollees of high SES in the same contracts (95% CI, 0.4-0.6 stars). Black enrollees had a 0.3-star lower simulated rating on average than White enrollees in the same contract (95% CI, 0.2-0.4 stars). For enrollees of low SES and Black enrollees, more of the disparity in star ratings could be attributed to within-contract differences (66% and 54%, respectively). Conversely, for Hispanic enrollees, the largest disparity was attributable to between-contract differences (85%). Within-contract differences for individual measures are presented in eTable 6 in the Supplement.

**Table 2.** Simulated Contract Star Ratings Stratified by Socioeconomic Status (SES) and Race/Ethnicity^a^

Group	Simulated rating, mean (SD)^b^	Difference (95% CI)^c^		Difference, %	
		Within contract	Between contract	Attributable to within contract	Attributable to between contract
SES group					
High SES	4.5 (0.7)	NA	NA	NA	NA
Low SES (either)^d^	4.2 (0.7)	0.3 (0.2 to 0.4)	0.3 (0.1 to 0.5)	53.0	47.0
Low SES (both)^d^	3.8 (0.8)	0.5 (0.4 to 0.6)	0.3 (0.1 to 0.4)	65.5	34.5
Race/ethnicity group					
White	4.4 (0.8)	NA	NA	NA	NA
Black	4.0 (0.8)	0.3 (0.2 to 0.4)	0.2 (0.1 to 0.3)	54.2	45.8
Hispanic	4.1 (0.8)	0.1 (−0.04 to 0.2)	0.4 (0.2 to 0.5)	16.0	84.0

Abbreviation: NA, not applicable.

^a^ All data are presented at the individual contract level (n = 454).

^b^ The simulated mean rating is the mean of the contract-level star ratings calculated only for members of each group without any risk adjustment. The SD corresponds to the mean rating. The within-contract difference represents the mean difference in the star rating between the low SES category and the high SES category, and between Black or Hispanic enrollees and White enrollees who are in the same contract. The between-contract difference represents the mean disparity that is attributable to enrollees of different groups being enrolled in plans of different quality.

^c^ The 95% CIs for the between-contract differences were calculated using a bootstrapped sample with 10 000 replications. The sum of the within-contract and between-contract differences may not add up to the overall difference in star ratings due to uneven enrollment of those with low SES and Black and Hispanic enrollees across contracts.

^d^ Low SES (either) refers to enrollees who have either low income or low educational attainment. Low SES (both) refers to those who have both low income and low educational attainment.

**Figure 1.**
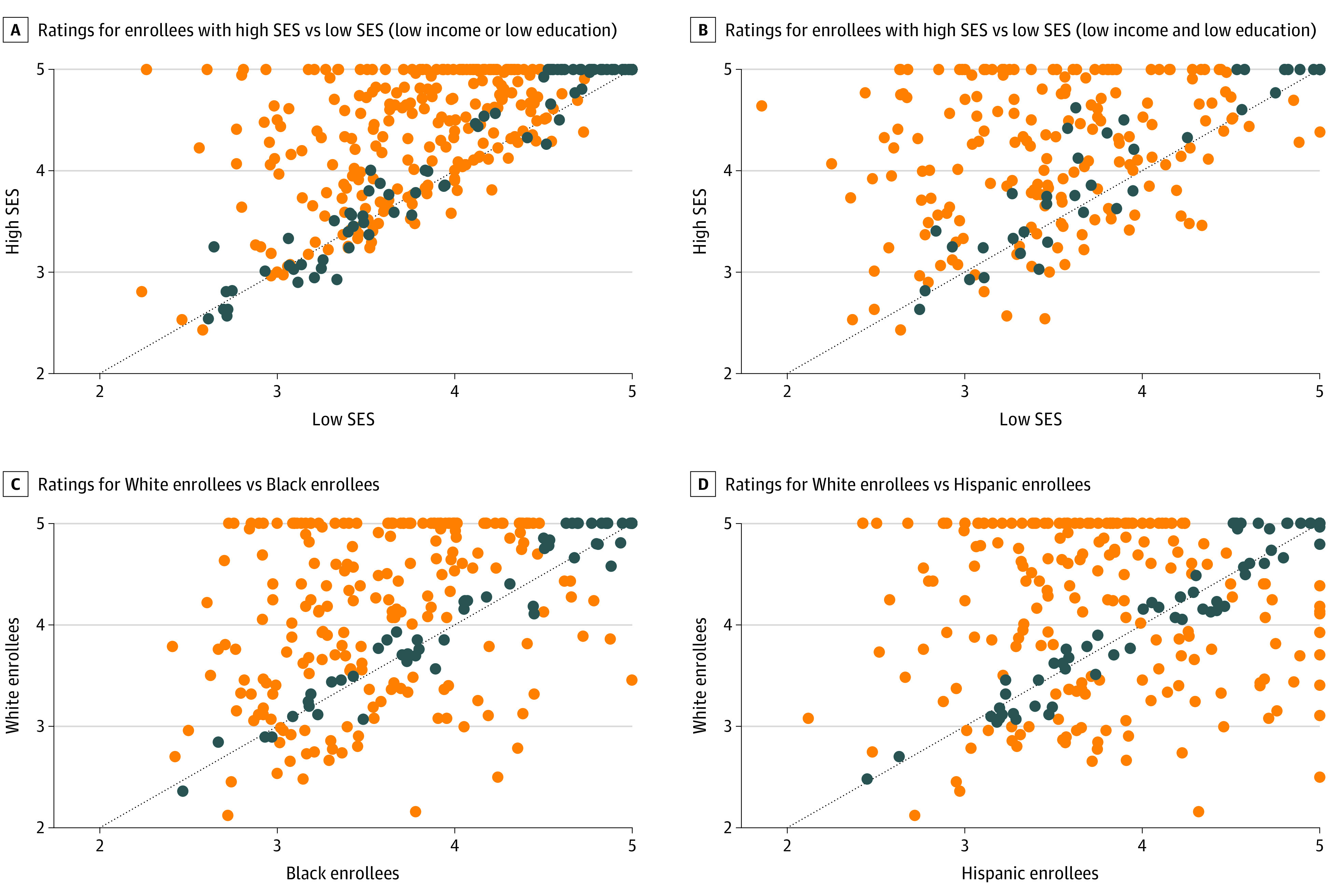
Association Between Simulated Star Ratings for Enrollees with High vs Low Socioeconomic Status (SES) and for White vs Racial/Ethnic Minority Enrollees in the Same Medicare Advantage Contract Each point represents a contract and plots the calculated star rating for each group. Dots above the 45-degree line indicate that either the high SES or the White enrollee stratified star ratings are higher than that of the comparison group. The US Centers for Medicare & Medicaid Services rounds star ratings to the nearest 0.5 increment. Dark blue dots represent that after rounding the star rating for each group would be the same. Orange dots represent that after rounding the stratified star ratings would be different.

Figure 1 shows the association between contract-level simulated star ratings for enrollees of high SES and low SES, as well as ratings for White and racial/ethnic minority enrollees. In 71% of contracts, enrollees of low SES had a lower stratified rating than those with high SES. The stratified star rating for Black enrollees was lower than the rating for White enrollees in 57% of contracts, and the rating for Hispanic enrollees was lower than that of White enrollees in 49% of contracts. The correlation coefficient between the star rating for enrollees of both low and high SES was 0.2 (95% CI, 0.03-0.4). The correlation coefficient between the rating for Black enrollees and the rating for White enrollees was 0.4 (95% CI, 0.3-0.5), and between Hispanic enrollees and White enrollees was 0.3 (95% CI, 0.2-0.4).

**Figure 2.**
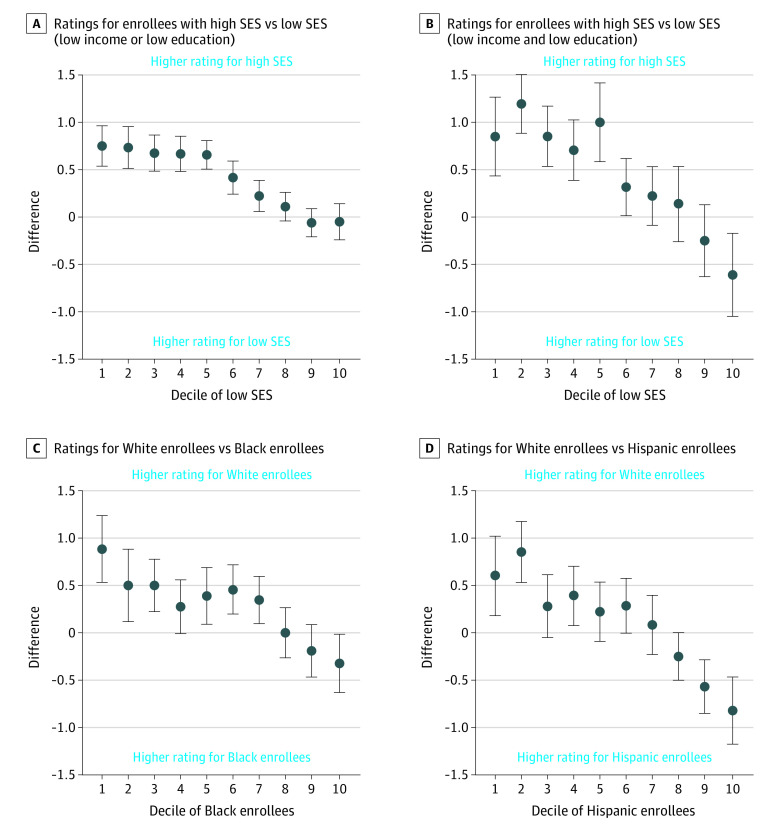
Socioeconomic and Racial/Ethnic Disparity in Simulated Star Rating by Decile of Low Socioeconomic Status (SES) or Race/Ethnicity Within the Medicare Advantage Contract Each point represents the disparity in star rating between each group (low SES and high SES or Black or Hispanic and White) by decile of SES or race/ethnicity concentration. Points above the 0 line indicate that the enrollees with low SES or Black or Hispanic enrollees in a contract perform worse in star-rating calculations than the comparison groups. If the 95% CIs do not cross the line at 0, then there is a statistically significant disparity at the .05 level. The cut points for each decile are included in eTable 2 in the Supplement.

Figure 2 compares the disparity in simulated star ratings between groups by decile of each group’s concentration. In contracts in the lowest decile of low-SES enrollment, enrollees of low SES had a 0.9-star lower rating than enrollees of high SES (95% CI, 0.4-1.3 stars; *P* < .001) compared with contracts in the highest decile of low-SES enrollment, where enrollees of low SES had a 0.6-star higher rating than enrollees of high SES (95% CI, 0.2-1.0 stars; *P* < .001). In contracts in the lowest decile of Black enrollment, Black enrollees had a 0.9-star lower rating than White enrollees (95% CI, 0.5-1.2 stars; *P* < .001) compared with contracts in the highest decile of Black enrollment, where Black enrollees had a 0.3-star higher rating than White enrollees (95% CI, −0.02 to 0.6 stars; *P* < .001). In contracts in the lowest decile of Hispanic enrollment, Hispanic enrollees had a 0.6-star lower rating than White enrollees (95% CI, 0.2-1.0 stars; *P* < .001) compared with contracts in the highest decile of Hispanic enrollment, where Hispanic enrollees had a 0.8-star higher rating than White enrollees (95% CI, 0.5-1.2 stars; *P* < .001). The stratified ratings for each group across deciles is presented in eFigure 1 in the Supplement. As the decile of low SES, Black, and Hispanic increases, so does the stratified rating for low SES, Black, and Hispanic enrollees. The cut points for each decile are included in eTable 2 in the Supplement.

**Figure 3.**
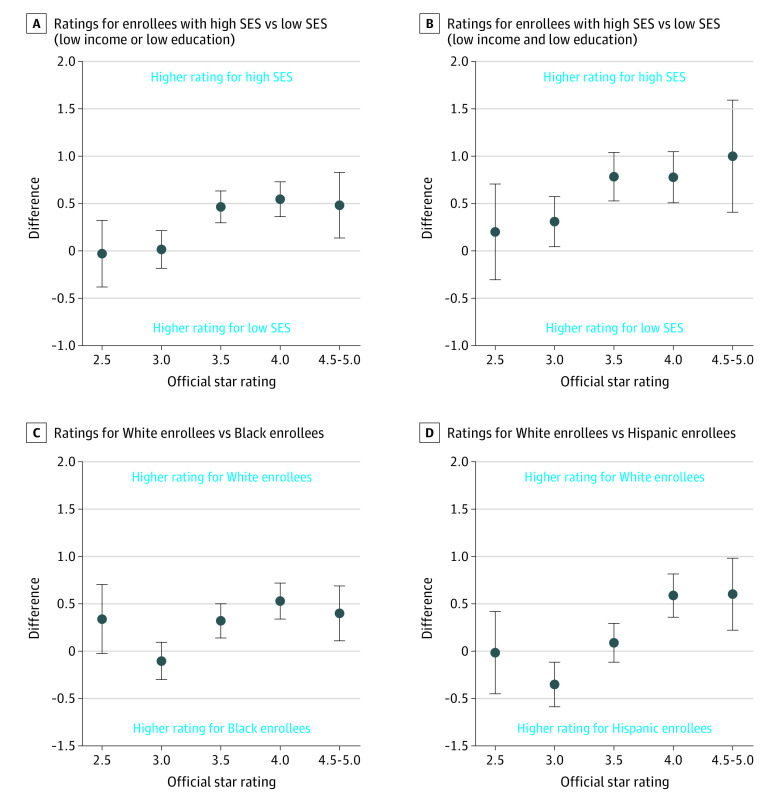
Socioeconomic and Racial/Ethnic Disparity in Simulated Star Ratings by US Centers for Medicare & Medicaid Services’ Published Contract-level Star Rating The y-axis in each panel represents either the average difference in stratified star rating between enrollees with low socioeconomic status (SES) and high SES or between White enrollees and Black or Hispanic enrollees. The x-axis represents the contract’s official star rating from 2016. Points above the 0 line indicate that the contracts perform worse for enrollees with low SES or Black and Hispanic enrollees. If the 95% CIs do not cross the line at 0, then there is a statistically significant disparity at the .05 level. For enrollees with low SES, the 4-star and 4.5- to 5-star–level disparities are significantly higher than the 2.5-star and 3-star groups (*P* < .05). For Black enrollees, the 4-star and 4.5- to 5-star–level disparities are significantly higher than the 3-star group (*P* < .05). For Hispanic enrollees, the 4-star and 4.5- to 5-star–level disparities are significantly higher than the 2.5-star and 3-star groups (*P* < .05).

Figure 3 plots the disparity in simulated star ratings by the official star rating assigned to each contract in 2015. In 4.5- to 5-star contracts, enrollees of low SES had a 1.0-star lower rating (95% CI, 0.4-1.6 stars) than enrollees of high SES. The disparity was not statistically significant in 2- to 2.5-star contracts (difference, 0.1; 95% CI, −0.4 to 0.7). Black enrollees had a 0.4-star lower rating (95% CI, 0.1-0.7 stars) than White enrollees within 4.5- to 5-star contracts and no statistical difference compared with White enrollees within 2.0- to 2.5-star contracts (difference, 0.3; 95% CI, −0.02 to 0.7). Hispanic enrollees had a 0.6-star lower rating (95% CI, 0.2-1.0 stars) in 4.5- to 5-star contracts and no statistical difference in 2- to 2.5-star contracts (difference, −0.01; 95% CI, −0.5 to 0.4). eFigure 2 in the Supplement presents the stratified ratings by official star rating, and the sensitivity analyses yielded similar results.

## Discussion

This cross-sectional study had 4 key findings. First, we observed only a modest correlation of simulated star ratings when calculated for enrollees of low SES and high SES, and between racial/ethnic minority enrollees and White enrollees in the same contract. Second, contracts with higher star ratings had larger racial/ethnic disparities than did those with lower star ratings. Third, the contracts with lower concentrations of individuals of low SES and Black or Hispanic individuals had larger disparities and worse quality for these individuals. Fourth, we identified both within-plan and between-plan disparities in the quality of care in the MA program, as measured by the star ratings.

We build on past work^3,10^ detailing disparities in the MA program in several key ways. First, we found that the disparities are evident not just in some selected outcomes,^3,10^ but across aggregate plan quality and in a composite metric (the star rating) that determines the distribution of $6 billion in annual bonus payments to MA plans.^27^ Second, to our knowledge, this is the first study to demonstrate that MA plans with higher-measured quality have larger magnitudes of disparity in quality within their enrolled populations. Third, we found a low correlation between a plan’s rating for its enrollees of low SES and Black and Hispanic enrollees, and its enrollees of high SES and White enrollees, although this may be largely due to the low reliability of the simulated scores.

There are many factors that could explain disparities in quality, including access to care, plan cultural competence, access to high-quality or racially concordant health care professionals,^28,29^ and other facets of structural racism. The CMS star ratings currently reward contracts that have greater disparities in quality for enrollees of low SES and Black or Hispanic enrollees. Without stratification, contracts may not be aware of their performance for racial/ethnic minorities and for persons with social risk factors. In an effort to avoid penalizing contracts that serve greater concentrations of individuals of low SES, in 2017 CMS implemented a Categorical Adjustment Index to adjust the calculation of star ratings based on the proportion of dual eligibility in a contract.^14^ This adjustment may make quality bonus payments more equitable across contracts, but it does not replace the benefit of stratification, which may help to better illustrate where disparities exist

This study is among the first, to our knowledge, that finds variation in disparities by some contract characteristics. We find that contracts with high concentrations of individuals of low SES and Black or Hispanic individuals actually perform better for those populations in stratified star ratings than they do for individuals of high SES and White individuals. It is possible that contracts that treat large numbers of these populations offer additional supplemental benefits or tailored interventions to better address their needs. They may also contract with insurance provider networks that perform better for these populations. Future work should seek to understand what other contract and plan-level factors may contribute to mitigate disparities in plans and the mechanisms that explain why disparities are larger in plans with higher star ratings and fewer racial/ethnic minority enrollees.

While we did find that certain contracts have larger disparities than others, these contracts may still be preferable to marginalized enrollees if they deliver better quality outcomes. However, we found that for contracts with higher proportions of Black or Hispanic enrollees and enrollees of low SES, the disparity both decreases and the absolute performance is better for Black or Hispanic enrollees and enrollees with low income (eFigure 1 in the Supplement). When comparing overall star ratings, higher-rated contracts do perform better for all enrollees; however, the disparities in care are larger, so the potential is not evident for all enrollees (eFigure 2 in the Supplement).

Several policy solutions could improve the measurement of quality in MA. First, CMS could publicly report on stratified star ratings, as we have herein. The CMS Office of Minority Health reports on disparities in measures across the entire MA program and provides stratified performance on specific measures at the contract level, but it does not publicly generate overall stratified star ratings or report on all outcomes on which contracts are evaluated.^30^ By publishing stratified star ratings, plans may have an incentive to improve the outcomes for their enrollees, and enrollees may also be able to make more informed decisions about which contracts will best serve them.^31^ While a plan may be able to take specific action on stratified individual measures, it may be separately beneficial to stratify aggregated star ratings. A stratified star rating may be more easily used by an enrollee seeking a plan that would most benefit them and may also do a better job of capturing the overall culture of health that a plan is producing than individual measures.

An alternative solution would be to formally include a measure of health equity in the calculation of star ratings,^32^ providing an increased overall rating to contracts that have lower disparities in care or that are able to reduce their disparities over time. This metric could be implemented in a similar fashion to current adjustments for plans that improve their overall quality, or included as a separate stand-alone measure incorporated into star ratings. An equity measure may create an incentive for a contract to reduce disparities without creating an incentive for plans to avoid enrollees who have social risk factors. The Medicare Payment Advisory Commission has suggested eliminating the 5-star rating system altogether, instead replacing it with a more parsimonious set of population-based measures.^27^ The present results highlight that any future form of MA quality measurement will likely require more focused attention on disparities.

### Limitations

This study has several limitations. First, we were unable to calculate ratings using the exact methods as CMS owing to restrictions in the years of available data and several measures that are not available to researchers. Our work here uses the CMS star system as a familiar method for summarizing quality information across measures and domains, but we do not present it as the only solution to the problem of measuring and incentivizing equitable care. Second, as a result of our exclusion criteria requiring at least 50 enrollees of each type to be included in an analysis, not all MA contracts were included in the final analysis (eTable 4 in the Supplement). Third, even with a requirement of including at least 50 enrollees in each contract for analysis, the sample sizes for some comparisons may be small (eTable 7 in the Supplement), which could lead to instability in our estimates. In our reliability analysis we found that some measures for some contracts may not have been reliable; however, when excluding these measures from our simulated ratings, the differences were minor. Fourth, Black and Hispanic enrollees had lower response rates to the CAHPS and HOS than White enrollees, though it is unclear if additional responses would widen or narrow the disparities we measured. Fifth, we intentionally combined Hispanic ethnicity and race into 1 variable to align with CMS’s current methodologies; however, these identities are multidimensional, and this approach may not have captured the experience of all beneficiaries. This study (eTable 3 in the Supplement) and others^33,34^ have found that there is often disagreement between self-report and CMS’s race/ethnicity measures, and they highlight the importance of using self-reported race/ethnicity data. Despite these limitations, this is the first effort that we are aware of to calculate star ratings stratified by self-reported enrollee type and to use these star ratings to demonstrate within-contract and between-contract differences.^21,35^

## Conclusions

Results of this cross-sectional study found that simulated star ratings for White enrollees and those with higher SES are only modestly associated with star ratings for minority enrollees and those with lower SES in the same contract. Contracts with higher Medicare star ratings have larger racial, ethnic, and socioeconomic disparities in quality. These findings indicate that the MA star ratings may need to be modified to explicitly consider and reward equity in care.

## Notes

Supplement. **eMethods.** Calculation of Star Ratings **eFigure 1.** Stratified star ratings by decile of low SES, black, and Hispanic **eFigure 2.** Stratified Star Ratings by Overall Rating **eTable 1.** List of quality measures included in stratified star rating calculation **eTable 2.** Distributions of contract level concentration of stratified groups **eTable 3.** Agreement between self-report and CMS race/ethnicity variables **eTable 4.** Enrollees and Contracts Remaining After Exclusion Criteria **eTable 5.** Comparison of response rates **eTable 6.** Within Contract Differences in Individual Measures **eTable 7.** Average Number of Observations included in Calculation by Measure and Race/Ethnicity **eTable 8.** Reliability of Measures by Strata
