# Supplementary material for: Association of Medicare Advantage Star Ratings With Racial, Ethnic, and Socioeconomic Disparities in Quality of Care
Source: JAMA Health Forum. 2021 Jun 11;2(6):e210793. doi: 10.1001/jamahealthforum.2021.0793 (PMC8796982; doi:10.1001/jamahealthforum.2021.0793)
Supplement: Supplement. — eMethods. Calculation of Star Ratings eFigure 1. Stratified star ratings by decile of low SES, black, and Hispanic eFigure 2. Stratified Star Ratings by Overall Rating eTable 1. List of quality measures included in stratified star rating calculation eTable 2. Distributions of contract level concentration of stratified groups eTable 3. Agreement between self-report and CMS race/ethnicity variables eTable 4. Enrollees and Contracts Remaining After Exclusion Criteria eTable 5. Comparison of response rates eTable 6. Within Contract Differences in Individual Measures eTable 7. Average Number of Observations included in Calculation by Measure and Race/Ethnicity eTable 8. Reliability of Measures by Strata [file jamahealthforum-e210793-s001.pdf]

Supplemental Online Content

Meyers DJ, Rahman M, Mor V, Wilson IB, Trivedi AN. Association of Medicare Advantage star ratings with racial, ethnic, and socioeconomic disparities in quality of care. *JAMA Health Forum*. 2021;2(6):e210793. doi:10.1001/jamahealthforum.2021.0793

eMethods. Calculation of Star Ratings

eFigure 1. Stratified star ratings by decile of low SES, black, and Hispanic

eFigure 2. Stratified Star Ratings by Overall Rating

eTable 1. List of quality measures included in stratified star rating calculation

eTable 2. Distributions of contract level concentration of stratified groups

eTable 3. Agreement between self-report and CMS race/ethnicity variables

eTable 4. Enrollees and Contracts Remaining After Exclusion Criteria

eTable 5. Comparison of response rates

eTable 6. Within Contract Differences in Individual Measures

eTable 7. Average Number of Observations included in Calculation by Measure and Race/Ethnicity

eTable 8. Reliability of Measures by Strata

This supplemental material has been provided by the authors to give readers additional information about their work.

© 2021 Meyers et al. *JAMA Health Forum.*

eMethods: Calculation of Star Ratings

CMS calculates star ratings for each contract on the basis of 30-35 measures of enrollee satisfaction and quality outcomes.[18] Each of these individual measures comes from HEDIS, CAHPS, HOS and other data reported by plans. Star ratings range from 2-5 stars in 0.5-star increments. While we could not calculate our stratified star ratings following the exact methodologies that CMS uses due to limitations in our data, we approximated their approach based on the 2015-star ratings. We first calculated ten individual measures from HEDIS data, five individual measures from the HOS, and six measures from CAHPS. Many of the CAHPS and HOS measures are indices which we also calculated following CMS methods. Finally, we also calculated disenrollment rates using the MBSF. Included measures are listed in Table S1. We could not calculate improvement measures as we did not have enough years of the HOS and CAHPS data, however the measures we did calculate account for ~70% of all measures used by CMS.

Next, we collapsed these individual measures from 2015 and 2016 to the contract level, and used cut-points used by CMS in 2015 to give each individual measure an assigned star value of 1-5. We aggregated these measures to the domain level, weighting each measure following CMS methods, and then aggregated to an overall Part C star rating. We first calculated these ratings for all enrollees in each contract. We then repeated the approach only including enrollees in each of our six stratified groups (high SES, either low income or low education, both low income and low education, white, black, and Hispanic). Similar to CMS, we then rounded our ratings to the nearest half-star increment. The approximated overall star rating we calculated was highly correlated to the official star rating CMS assigned to each contract (correlation coefficient=0.89, mean difference 0.1 stars).

As we combined data from across multiple years, we included a contract in our analysis if they had at least 50 enrollees we could calculate measures for across either year. If a contract only had one year of data in either 2015 or 2016, we would only include that year in our calculation if the sample allowed. We required a contract to have individual measures successfully calculated for at least 75% of component measures to be assigned a star rating for any comparison.

© 2021 Meyers et al. *JAMA Health Forum.*

eFigure 1: Stratified star ratings by decile of low SES, black, and Hispanic

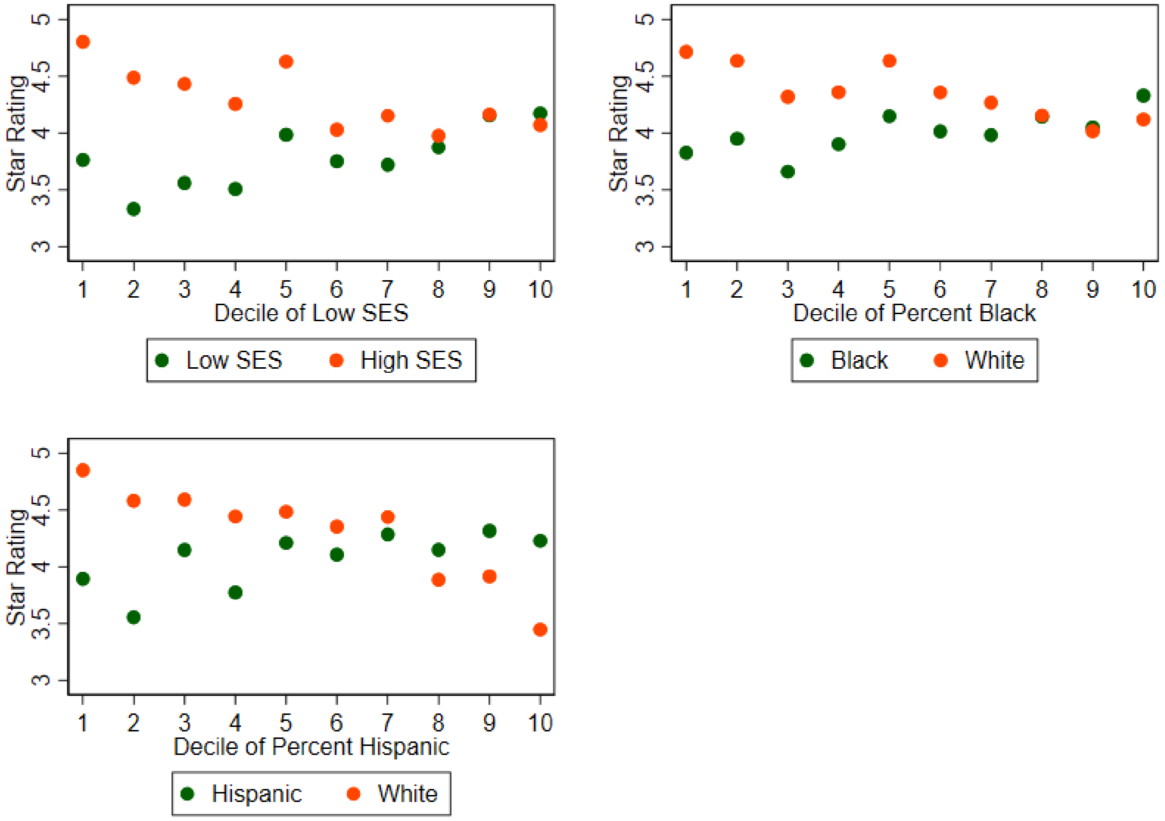

Notes: The y-axis represents the calculated star rating stratified by group. The x-axis for each panel is the decile of concentration for each stratified group.

© 2021 Meyers et al. *JAMA Health Forum.*

eFigure 2: Stratified Star Ratings by Overall Rating

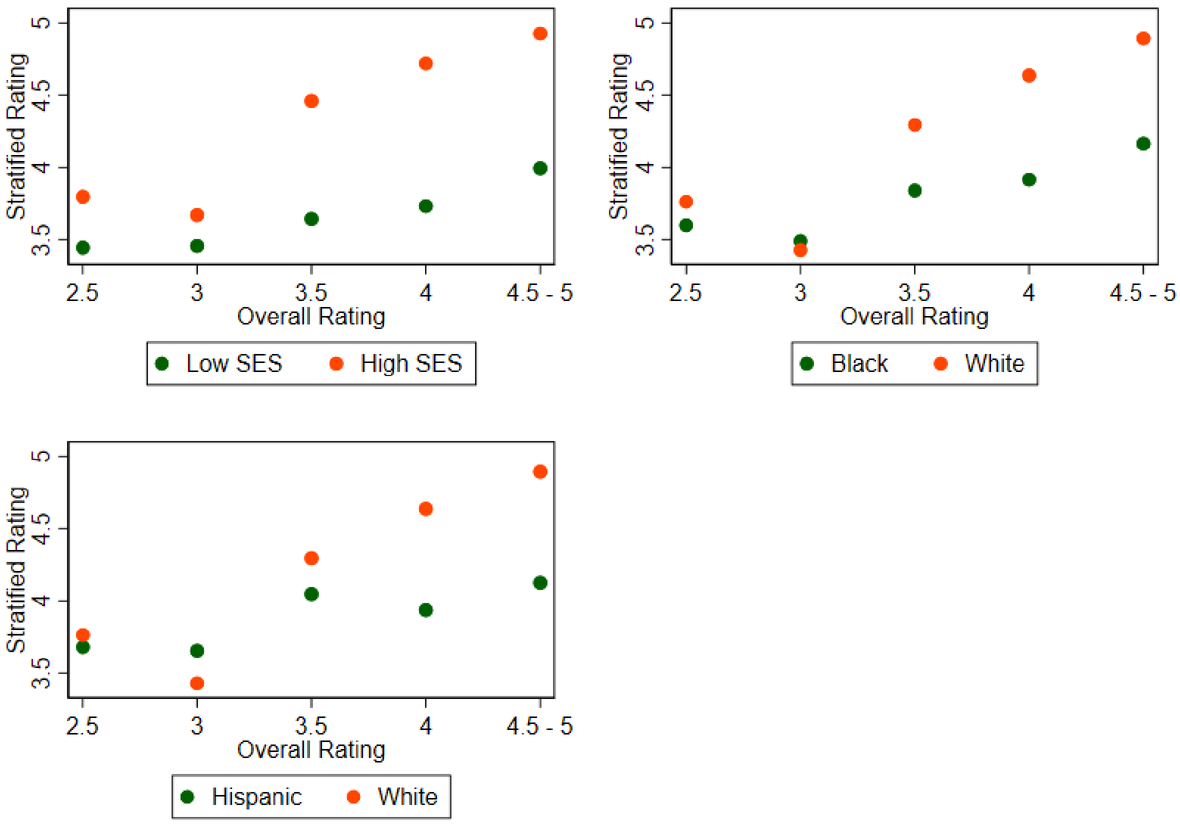

Notes: The y-axis represents the simulated star rating stratified by group. The x-axis for each panel is the official star rating.

© 2021 Meyers et al. *JAMA Health Forum.*

eTable 1: List of quality measures included in stratified star rating calculation

Measure	Source	Included in Analysis	Reason for Exclusion
Measure: C01 - Colorectal Cancer Screening	HEDIS	Yes	
Measure: C02 - Cardiovascular Care – Cholesterol Screening	HEDIS	No	Data not included in our HEDIS files
Measure: C03 - Diabetes Care – Cholesterol Screening	HEDIS	No	Data not included in our HEDIS files
Measure: C04 - Annual Flu Vaccine	CAHPS	No	Data not included in our CAHPS files
Measure: C05 - Improving or Maintaining Physical Health	HOS	Yes	
Measure: C06 - Improving or Maintaining Mental Health	HOS	Yes	
Measure: C07 - Monitoring Physical Activity	HOS	Yes	
Measure: C08 - Adult BMI Assessment	HEDIS	Yes	
Measure: C09 - Special Needs Plan (SNP) Care Management	Plan reporting	No	Plan reported data not available to researchers
Measure: C10 - Care for Older Adults – Medication Review	HEDIS	No	Data not included in our HEDIS files
Measure: C11 - Care for Older Adults – Functional Status Assessment	HEDIS	No	Data not included in our HEDIS files

© 2021 Meyers et al. *JAMA Health Forum.*

Measure: C12 - Care for Older Adults – Pain Assessment	HEDIS	No	Data not included in our HEDIS files
Measure: C13 - Osteoporosis Management in Women who had a Fracture	HEDIS	Yes	
Measure: C14 – Diabetes Care – Eye Exam	HEDIS	Yes	
Measure: C15 – Diabetes Care – Kidney Disease Monitoring	HEDIS	Yes	
Measure: C16 - Diabetes Care – Blood Sugar Controlled	HEDIS	Yes	
Measure: C17 - Diabetes Care – Cholesterol Controlled	HEDIS	Yes	
Measure: C18 - Controlling Blood Pressure	HEDIS	Yes	
Measure: C19 – Rheumatoid Arthritis Management	HEDIS	Yes	
Measure: C20 – Improving Bladder Control	HOS	Yes	
Measure: C21 - Reducing the Risk of Falling	HOS	Yes	
Measure: C22 – Plan All-Cause Readmissions	HEDIS	Yes	
Measure: C23 - Getting Needed Care	CAHPS	Yes	
Measure: C24 - Getting Appointments and Care Quickly	CAHPS	Yes	
Measure: C25 - Customer Service	CAHPS	Yes	
Measure: C26 - Rating of Health Care Quality	CAHPS	Yes	
Measure: C27 - Rating of Health Plan	CAHPS	Yes	
Measure: C28 - Care Coordination	CAHPS	Yes	
Measure: C29 - Complaints about the Health Plan	CMS	No	CMS data source not available to researchers
Measure: C30 - Members Choosing to Leave the Plan	MBSF	Yes	
Measure: C31 - Health Plan Quality Improvement	CMS	No	Not possible with our methodology
Measure: C32 - Plan Makes Timely Decisions about Appeals	CMS	No	CMS data source not available to

© 2021 Meyers et al. *JAMA Health Forum.*

			researchers
Measure: C33 - Reviewing Appeals Decisions	CMS	No	CMS data source not available to researchers

© 2021 Meyers et al. *JAMA Health Forum.*

eTable 2: Distributions of contract level concentration of stratified groups

	Low Income and Low Education			Black			Hispanic		
Mean	8.8%			18.9%			24.0%		
Median	8.3%			14.3%			15.9%		
Decile	Median	Lower Bound	Upper Bound	Median	Lower Bound	Upper Bound	Median	Lower Bound	Upper Bound
1	2.3%	1.4%	3.0%	3.4%	2.0%	4.2%	2.7%	1.6%	3.6%
2	3.5%	3.0%	3.9%	5.0%	4.3%	6.2%	4.4%	3.6%	5.1%
3	4.5%	4.0%	5.1%	7.3%	6.2%	8.3%	6.2%	5.1%	7.5%
4	5.9%	5.3%	6.6%	9.6%	8.3%	10.9%	9.1%	7.6%	11.5%
5	7.3%	6.6%	8.3%	12.5%	11.0%	14.3%	13.5%	11.6%	15.9%
6	9.0%	8.3%	9.6%	15.7%	14.4%	16.8%	18.2%	15.9%	20.9%
7	10.4%	9.7%	11.0%	19.6%	17.0%	22.1%	24.0%	21.2%	27.5%
8	12.1%	11.1%	13.5%	25.5%	22.5%	28.1%	34.7%	27.9%	39.6%
9	14.7%	13.5%	16.3%	35.7%	28.2%	41.5%	47.5%	39.7%	59.4%
10	19.2%	16.4%	21.9%	55.2%	42.4%	82.8%	82.5%	59.8%	100.0%

© 2021 Meyers et al. *JAMA Health Forum.*

eTable 3: Agreement between self-report and CMS race/ethnicity variables

	HOS and MBSF		HOS and RTI		CAHPS and MBSF		CAHPS and RTI	
	Sensitivity	Specificity	Sensitivity	Specificity	Sensitivity	Specificity	Sensitivity	Specificity
White	97.4	64.6	87.1	73.2	95.5	92.6	89.4	97
Black	96.0	91.2	95.5	91.7	93.2	99.6	91.8	99.8
Hispanic	29.0	97.1	85.8	95.0	36.5	90.7	87.2	81.6

Notes: The MBSF race is the race recorded for each enrollee in CMS data which comes originally from the social security administration. The RTI race code applies a predictive algorithm to classify enrollees in race/ethnicity categories. Both are available in the Master Beneficiary Summary File. In both the MBSF variables, Hispanic is considered an exclusive race category. In the HOS and the CAHPS, Hispanic ethnicity is asked as a separate question from race. For this table, we coded the HOS and CAHPS variables in the same way as the MBSF, considering someone to be Hispanic, or any other race non-Hispanic. We then compared the self-report as a gold standard to the two MBSF variables.

© 2021 Meyers et al. *JAMA Health Forum.*

eTable 4: Enrollees and Contracts Remaining After Exclusion Criteria

	Enrollees						Contracts						
	All Enrollees	High SES Enrollees	Both Low Income and Education	White	Black	Hispanic	N of contracts	% Contracts	2-2.5 Stars	3 Stars	3.5 Stars	4 Stars	4.5 to 5 Stars
50+ High SES and Low SES	67%	55%	93%	58%	84%	89%	264	58%	81%	93%	61%	58%	39%
50+ White and Black Enrollees	85%	82%	91%	84%	98%	81%	357	78%	89%	90%	73%	87%	78%
50+ White and Hispanic Enrollees	83%	80%	88%	81%	88%	83%	333	73%	78%	81%	79%	81%	72%

Notes: This table presents the percent of enrollees and contracts of different official star ratings that are included in each of our analyses. To ensure the stability of our estimates, we required that at least 50 enrollees of a type were included for each comparison. Each percentage in the enrollee columns are the percent of all enrollees of that type sampled in the HOS or CAHPS that are included in the sample after each exclusion. The contract columns represent he % of all MA contracts of a given star rating that are included in that sample.

© 2021 Meyers et al. *JAMA Health Forum.*

eTable 5: Comparison of response rates

Individual Level Response Rates by Enrollee Characteristic

	Source	Overall	Not Low Income	Low Income	White	Black	Hispanic
Response Rate	CAHPS	38.6	41.8	32.5	43.2	34.2	30.8
	HOS	43.9	45.0	41.4	44.9	40.7	38.3

Contract Level Response Rates by Overall Rating

	Source	Overall	2-2.5	3	3.5	4	4.5-5	Unrated
Response Rate	CAHPS	38.2	39.5	35	39.7	40.1	46.2	32.8
	HOS	43.5	43.7	41.2	43.9	45	46.8	41.8

Contract Level Response Rates by Decile of Enrollment

Decile of:		1	2	3	4	5	6	7	8	9	10
Low SES	CAHPS	41%	38%	39%	37%	38%	35%	35%	33%	34%	35%
	HOS	45%	44%	44%	45%	47%	41%	42%	42%	42%	43%
Black	CAHPS	43%	41%	39%	38%	38%	36%	35%	36%	36%	34%
	HOS	46%	46%	44%	45%	43%	43%	43%	42%	43%	40%
Hispanic	CAHPS	43%	43%	39%	39%	38%	36%	36%	35%	34%	34%
	HOS	46%	47%	45%	44%	43%	42%	40%	42%	42%	44%

© 2021 Meyers et al. *JAMA Health Forum.*

eTable 6: Within Contract Differences in Individual Measures

Measure	Difference from White			
	Black	p-value	Hispanic	p-value
Rheumatoid Arthritis Management	0.5%	0.641	2.7%	0.021
Diabetes Care – Kidney Disease Monitoring	0.8%	0.003	1.0%	0.001
Diabetes Care – Eye Exam	1.8%	0.004	4.2%	0
Plan All-Cause Readmissions	0.5%	0.078	-1.5%	0
Improving Bladder Control	0.0%	0.984	3.1%	0
Colorectal Cancer Screening	3.8%	0	3.2%	0
Adult BMI Assessment	0.5%	0.238	1.5%	0
Monitoring Physical Activity	6.6%	0	7.5%	0
Osteoporosis Management in Women who had a Fracture	0.6%	0.805	5.0%	0.017
Diabetes Care – Blood Sugar Controlled	-0.5%	0.112	1.5%	0
Diabetes Care – Cholesterol Controlled	-2.3%	0	-2.8%	0
Controlling Blood Pressure	-8.2%	0	0.6%	0.419
Getting Needed Care	-2.9	0	-1.2	0
Getting Appointments and Care Quickly	-3.4	0	-3.0	0
Rating of Health Plan	6.7	0	26.4	0
Rating of Health Care Quality	-24.0	0	-3.4	0.032
Care Coordination	0.2	0.199	-1.3	0
Customer Service	-0.1	0.794	-1.0	0.001
Members Choosing to Leave the Plan	0.7%	0	1.2%	0
Reducing the Risk of Falling	1.6%	0	2.9%	0
Improving or Maintaining Physical Health	3.6%	0.011	4.4%	0.008
Improving or Maintaining Mental Health	0.8%	0.577	-3.0%	0.073

Notes: This table displays within contract differences in outcomes for each of the measures included in the study. The Black column shows the difference in outcome for Black enrollees from White enrollees, and the Hispanic column shows the difference between the outcomes for Hispanic enrollees and White enrollees.

© 2021 Meyers et al. *JAMA Health Forum.*

eTable 7: Average Number of Observations included in Calculation by Measure and Race/Ethnicity

	White				Black				Hispanic			
Variable	Mean Sample Size	Std. Dev.	Min	Max	Mean Sample Size	Std. Dev.	Min	Max	Mean Sample Size	Std. Dev.	Min	Max
Rheumatoid Arthritis Management	72.7	26.3	51	172	67.3	15.7	55	85	62.9	9.4	51	79
Diabetes Care – Kidney Disease Monitoring	139.1	82.9	51	535	91.2	35.7	52	205	134.3	104.9	51	428
Diabetes Care – Eye Exam	128.3	71.9	51	535	89.8	34.9	52	205	127.7	100.7	51	511
Plan All-Cause Readmissions	294.5	214.2	53	1724	124.0	99.3	51	799	148.2	96.8	51	418
Improving Bladder Control	242.4	147.4	54	1524	98.5	48.3	51	283	139.5	111.0	51	645
Colorectal Cancer Screening	209.6	343.6	51	2554	105.7	66.8	51	397	139.3	116.1	51	696
Adult BMI Assessment	168.1	233.6	52	2345	96.4	50.8	51	255	125.6	112.5	51	607
Monitoring Physical Activity	615.8	414.1	52	3996	185.2	145.0	51	891	269.9	290.1	52	1727
Diabetes Care – Blood Sugar Controlled	139.2	78.7	52	566	92.0	42.0	51	259	122.8	88.1	53	395
Diabetes Care – Cholesterol Controlled	139.2	78.7	52	566	92.0	42.0	51	259	122.8	88.1	53	395
Controlling Blood Pressure	153.1	96.2	51	654	108.2	59.6	51	292	120.9	85.6	53	401
Getting Needed Care	350.2	306.1	51	2468	114.3	91.6	51	686	144.0	105.9	51	570
Getting Appointments and Care Quickly	447.7	402.8	55	3332	145.7	144.9	51	1269	179.9	129.1	51	643
Rating of Health Plan	482.1	438.4	51	3534	152.6	150.3	51	1317	196.8	151.4	53	773
Rating of Health Care Quality	487.4	446.0	52	3662	154.4	154.9	51	1377	197.8	152.6	51	761
Members Choosing to Leave the Plan	2135.9	1624.7	52	12711	527.6	575.8	51	5281	660.7	888.9	51	5233
Care Coordination	422.6	380.3	52	3156	143.9	137.6	51	1191	181.6	134.8	54	693
Customer Service	209.7	168.9	51	1320	104.8	80.2	51	556	129.8	72.7	51	359
Reducing the Risk of Falling	289.7	182.2	52	1930	127.0	79.2	51	481	191.5	184.7	53	1058

© 2021 Meyers et al. *JAMA Health Forum.*

Notes: This table includes the mean, standard deviation, min and max of the number of observations included for each measure at the plan level, stratified by race/ethnicity.

© 2021 Meyers et al. *JAMA Health Forum.*

eTable 8: Reliability of Measures by Strata

	White		Black		Hispanic		Low SES	
Variable	Mean Reliability	n <0.6	Mean Reliability	n <0.6	Mean Reliability	n <0.6	Mean Reliability	n <0.6
Rheumatoid Arthritis Management	0.72	0	0.67	0	0.77	0	n/a	n/a
Diabetes Care – Kidney Disease Monitoring	0.67	62	0.60	50	0.82	0	0.76	1
Diabetes Care – Eye Exam	0.88	0	0.85	0	0.87	0	0.81	0
Plan All-Cause Readmissions	0.72	50	0.63	80	0.70	29	0.57	50
Improving Bladder Control	0.66	95	0.63	37	0.69	18	0.69	21
Colorectal Cancer Screening	0.90	0	0.86	0	0.88	0	0.82	0
Adult BMI Assessment	0.94	0	0.93	0	0.91	0	0.86	0
Monitoring Physical Activity	0.83	4	0.67	61	0.70	28	0.76	0
Diabetes Care – Blood Sugar Controlled	0.74	0	0.70	0	0.73	0	0.67	0
Diabetes Care – Cholesterol Controlled	0.89	0	0.84	0	0.88	0	0.84	0
Controlling Blood Pressure	0.91	0	0.89	0	0.91	0	0.89	0
Reducing the Risk of Falling	0.76	4	0.65	39	0.73	0	0.67	13
Members Choosing to Leave the Plan	0.99	0	0.99	0	0.98	0	0.98	0
Care Coordination	0.87	0	0.65	36	0.79	0	0.70	0
Customer Service	0.84	0	0.66	4	0.72	0	0.61	42
Getting Needed Care	0.91	0	0.72	0	0.79	0	0.72	0
Getting Appointments and Care Quickly	0.91	0	0.74	0	0.78	0	0.71	0
Rating of Health Plan	0.92	0	0.76	0	0.83	0	0.75	0
Rating of Health Care Quality	0.90	0	0.69	0	0.80	0	0.71	0

Notes: This table includes the mean reliabilities for each measure by strata. It also includes the number of contracts with greater than 50 observations for that measure-strata that have a reliability under 0.6 which is a cutoff CMS uses to suppress unreliable measures. The reliabilities are interrater reliabilities calculated from a random effects model. There was no reliability

© 2021 Meyers et al. *JAMA Health Forum.*

calculated for the low SES category as there were too few contracts that had at least 50 observations on that measure for that strata.

© 2021 Meyers et al. *JAMA Health Forum.*
